# Optimization of culture read times using full microbiology laboratory automation

**DOI:** 10.1128/jcm.00232-26

**Published:** 2026-09-18

**Authors:** Janiece S. Glover, Bailey B. Demarest, Pam J. Foster, Kendall A. Bryant

**Affiliations:** 1 Department of Pathology, Microbiology, and Immunology, Vanderbilt University Medical Center 12328, Nashville, Tennessee, USA; Endeavor Health, Evanston, Illinois, USA

**Keywords:** microbiology automation

## Abstract

Clinical microbiology laboratories are increasingly adopting automation to meet testing demands, improve throughput, and accelerate diagnostic turnaround. This study evaluated optimal culture read times using Copan WaspLab microbiology laboratory automation (MLA) to determine if earlier read times affect accuracy and turnaround times as they pertain to microbial identification and antimicrobial susceptibility testing (AST). A total of 374 patient specimens were cultured simultaneously using a conventional incubation method and MLA, with MLA images captured from 10 to 72 h of incubation. Non-urine specimens were ready for workup in over 50% of cases at 16 h, 92% by 24 h, and 100% by 36 h, while 90% of urine cultures were ready for workup by 16 h. Using frozen isolates (127 gram-positive bacteria, 83 gram-negative bacteria, and 12 yeasts), we further evaluated the accuracy of MALDI-TOF identification and AST results for cultures incubated for 10 h and 16 h in MLA. Identification accuracy remained high across all groups, with gram-negative bacteria achieving 98.8% accuracy at 10 h and 97.6% at 16 h, gram-positive bacteria showing 97.6% accuracy at 10 h and 98.4% at 16 h, and yeasts demonstrating 91.6% accuracy at 10 h and 100% at 16 h of incubation, respectively. AST results using cultures at both time points demonstrated essential agreement above 90% for most drugs on Vitek 2 GN-807N, GP75, and ST02 AST cards. Minor and major errors were rare; however, elevated error rates were observed for specific drugs (e.g., cefazolin, piperacillin/tazobactam, nitrofurantoin, and linezolid), largely due to a limited number of susceptible isolates and breakpoint-related challenges. MLA enables earlier culture reads while maintaining ID and AST accuracy.

**IMPORTANCE:**

Microbiology laboratory automation (MLA) is increasingly used to address rising test volumes and workforce limitations; however, optimal incubation durations for automated workflows are not well defined. In this study, we demonstrate that cultures from a range of specimen types can be reliably read as early as 16 h and finalized by 36 h without compromising organism identification or antimicrobial susceptibility testing accuracy compared to conventional incubation. These findings support the use of earlier culture read time points in MLA workflows, enabling faster turnaround of clinically actionable results. Implementation of optimized incubation times has the potential to improve laboratory efficiency and accelerate patient care decisions while maintaining diagnostic reliability.

## INTRODUCTION

The adoption of microbiology laboratory automation (MLA) has increased due to rising microbiology test volumes, a shortage of trained laboratory scientists, and consolidation of laboratory testing in many healthcare systems (1, 2). MLA enhances laboratory efficiency and reduces the manual labor needed for culture processing and analysis by automating specimen plating and allowing digital plate reading (3, 4). Most MLA systems have smart incubators that offer consistent incubation environments, which promote faster colony growth (5). Reductions in incubation times based on early growth observations obtained using MLA systems have been documented for urine and blood culture specimens (6–9), while very little data are available for other specimen types.

Currently, the incubation durations using conventional incubation (CI) systems are not specified in the Clinical Microbiology Procedures Handbook or the Manual of Clinical Microbiology (10, 11), and only limited guidance is provided in other best practice documents such as the American Society for Microbiology’s Cumitechs for certain culture types (12, 13). These guidance documents only suggest that plate readings should be performed “once daily,” resulting in variable culture incubation durations across laboratories due to differences in workflow (13–17). Manufacturers of MLA systems do not provide specific recommendations for incubation durations, primarily due to the limited data available on optimal minimum or maximum incubation times for these systems. The lack of guidance for standardized incubation durations presents challenges for manufacturers of identification and antimicrobial susceptibility test (AST) systems, leading to varying recommendations for minimum and maximum incubation times. Moreover, there is no mention of alternative incubation durations specifically tailored for MLA. This gap in knowledge in standardized incubation duration complicates workflow optimization, especially as laboratories increasingly adopt MLA to improve efficiency (18).

In the clinical microbiology laboratory, instruments that automate and accelerate time to results are essential for improving laboratory efficiency and patient care (19, 20). The introduction of MALDI-TOF mass spectrometry (MS) for rapid organism identification and the use of automated AST systems are the cornerstones of the modern clinical microbiology laboratory (21–24). However, MALDI-TOF MS systems recommend at least 18–24 h of incubation for gram-negative and gram-positive bacteria to achieve accurate identification (25, 26). Additionally, automated AST instruments provide varying manufacturer-recommended incubation durations, ranging from 10 h to 24 h depending on the Gram reactivity of the organism (27–29). None of the commercially available MALDI-TOF MS or AST systems currently accommodate reduced incubation durations with the use of MLA systems. There are limited data in the literature on the reliability of MALDI-TOF MS identification and AST from cultures at earlier time points than specified by the manufacturer for either MLA or CI systems (30–34).

In this study, we optimized the plate reading times for common culture types using Copan WASPLab MLA, compared with standard reading times using conventional incubation systems. Additionally, we evaluated the reliability of the Vitek Prime MALDI-TOF MS for identifying clinically relevant microbes and assessed the performance of Vitek 2 automated AST systems for performing susceptibility testing using cultures with shorter incubation durations in the MLA system.

## MATERIALS AND METHODS

### Specimens for optimizing incubation duration on MLA

A total of 378 residual patient specimens included 110 from sterile sources (body fluids, joints, and tissues), 49 from abscesses, 43 from deep wounds, 44 from superficial wounds, 28 from respiratory sources, and 104 urine samples were used for the study. The specimens were stored refrigerated at 4°C for up to 5 days before being used in the study. An additional 22 spiked respiratory specimens were added to the study due to a limited number of common pathogens isolated from the respiratory specimens described above. Spiked samples were prepared by adding 1 μL of a 0.5 McFarland suspension of common respiratory pathogens to residual respiratory specimens collected in Snot Buster vials (Copan Diagnostics, Inc., Murrieta, CA, USA), which yielded approximately 150 CFU/μL of the spiked organism in the sample. A 10 μL loop was used to inoculate plates, resulting in approximately 1,500 CFUs per plate (Table 1).

**TABLE 1:** Ready-to-read cultures at different incubation times on WASPLab

	Time point, no. (%) of samples								
Urine samples									
Hours	10	12	14	16	18	24	36	72	Total
RTR (% RTR)	0	4 (7)	42 (82)	5 (91)	5 (100)				55
Sterile sites									
Hours	10	12	14	16	18	24	36	72	Total
RTR (% RTR)	1 (4)	4 (20)	4 (36)	6 (60)	6 (84)	2 (92)	2 (100)	0 (100)	25
Abscess samples									
Hours	10	12	14	16	18	24	36	72	Total
RTR (% RTR)	0	0	8 (27)	7 (50)	7 (73)	5 (90)	3 (100)	0 (100)	30
Superficial wound samples									
Hours	10	12	14	16	18	24	36	72	Total
RTR (% RTR)	0	0	2 (9)	11 (59)	5 (82)	3 (100)	0 (100)	0 (100)	22
Deep wound samples									
Hours	10	12	14	16	18	24	36	72	Total
RTR (% RTR)	0	1 (4)	6 (28)	6 (52)	6 (76)	2 (100)	0 (100)	0 (100)	25
Respiratory samples									
Hours	10	12	14	16	18	24	36	72	Total
RTR (% RTR)	0	3 (10)	13 (45)	9 (76)	4 (90)	2 (97)	1 (100)	0 (100)	29
All sites combined, excluding urine samples									
Hours	10	12	14	16	18	24	36	72	Total
RTR (% RTR)	1 (1)	8 (8)	22 (26)	39 (58)	28 (81)	8 (96)	6 (100)	0 (100)	122

#### Procedure for bacterial culture using MLA system

Non-urine specimens were plated with a 10 µL loop using the WASP system onto Trypticase Soy Agar with 5% sheep blood, Chocolate II agar, and MacConkey II agar (BD, Sparks, MD, USA) and incubated within the MLA system at 35 °C with 5% CO₂. Digital culture images were captured by WASPLab at 10, 12, 14, 16, 18, 20, 22, 24, 36, and 72 h of incubation.

Urine specimens were plated using the WASP 1 µL loop onto a biplate containing CHROMagar Orientation and Trypticase Soy Agar with 5% sheep blood (BD, Sparks, MD, USA) and incubated within the MLA system at 35°C under ambient atmospheric conditions. Digital images were captured at 10, 12, 14, 16, 18, 20, 22, and 24 h of incubation. Culture interpretation at all time points was performed using WASPLab images.

#### Procedure for bacterial culture using CI system

All non-urine specimens were plated manually using a 10 µL loop onto the same plates as MLA and incubated in CI at 35°C with and without 5% CO_2_ according to standard laboratory protocol. Non-urine cultures were read manually at 24 h, 48 h, and 72 h time points of incubation. Urine samples were plated manually using a 1 µL loop onto the biplate and incubated at 35°C without 5% CO_2_.

### Bacterial culture evaluation for the optimization study

Digital culture images from the MLA were read by technologists who determined when the culture was ready to read (RTR), which was defined as enough individual colonies available for MALDI-TOF MS spotting and AST setup following the manufacturer’s recommended protocol for Vitek MS Prime (bioMérieux, Inc., Durham, NC, USA) and Vitek 2 AST (bioMérieux, Inc., Durham, NC, USA). Results interpreted from digital images from the MLA were compared to results of the standard manual “in-hand” culture reading methods for identification of organisms. A discordant result between MLA and CI was defined as a significant pathogen discordance between the MLA and CI results. A quantitation discordant was defined as a difference of two or more quantitation units between the CI and MLA cultures. Appearance of single colonies or normal flora breakthrough were not counted as discordant in the study. The accuracy of MLA was calculated as the percentage of cultures with discordant results compared to the CI culture. The original patient care results were plated upon receipt of the specimen using the same media and using CI methods. These patient care results were examined in all discordant cases but were not used in the accuracy calculations.

### Bacterial isolates for MALDI-TOF MS identification and AST evaluation

A total of 222 previously frozen clinical isolates were analyzed for the MALDI-TOF MS identification study, comprising 127 gram-positive bacteria, 83 gram-negative bacteria, and 12 yeast species. These isolates represented a diverse range of clinically relevant organisms to evaluate MALDI-TOF MS performance across multiple taxa (Table S1).

For the AST evaluation, clinical isolates representing gram-negative and gram-positive organisms were included. The Vitek GN-807N Card (bioMérieux, Inc., Durham, NC, USA) was used for assessing AST at early time points on MLA using 50 gram-negative isolates (35 Enterobacterales, 8 *Pseudomonas aeruginosa*, 7 *Acinetobacter* spp.), which included 13 carbapenem-resistant organisms and 7 ESBL-producing organisms. The Vitek AST-GP75 Card (bioMérieux, Inc., Durham, NC, USA) was used for testing AST at early time points on MLA using 49 gram-positive isolates (20 *Enterococcus* spp., 18 *Staphylococcus aureus*, 11 coagulase-negative staphylococci). The Vitek AST-ST02 Card (bioMérieux, Inc., Durham, NC, USA) was used to evaluate AST of 52 streptococcal isolates at early time points on MLA including 13 β-hemolytic streptococci, 14 *Streptococcus anginosus* group, 5 *Streptococcus dysgalactiae* complex, 1 *Streptococcus equinus* group, 15 *Streptococcus mitis* group (including 9 *Streptococcus pneumoniae*), and 4 *Streptococcus salivarius* group.

### Procedures for Vitek MS prime identification and Vitek 2 AST at early MLA time points

Bacteria were evaluated by subculturing frozen stocks twice to BD BBL Trypticase Soy Agar 5% Sheep Blood with 24 h incubation time for each subculture. A 0.5 McFarland suspension was prepared from fresh colonies and plated using WASP with subsequent incubation in the MLA. Parallel control plates were also manually plated and incubated in CI using the same suspension. A separate blood agar plate was used for the 10 h and 16 h MALDI-TOF identifications to minimize the time plates were outside of the WASPLab.

MLA cultures incubated for 10 h and 16 h were identified by Vitek MS Prime per manufacturer’s protocol. Earlier MLA incubation time points were compared with CI cultures incubated for 18–24 h and evaluated using standard laboratory workflows. The accuracy of MALDI-TOF identification was calculated as the proportion of isolates correctly identified at the species level as compared to CI culture. Concordance and discrepancies were recorded for each organism group (gram-negative, gram-positive, and yeast).

AST of organisms from MLA cultures at 10 h and 16 h time points were compared to CI cultures incubated for 18–24 h using appropriate Vitek 2 AST cards. AST essential agreement (EA) was calculated as the proportion of isolates where the minimum inhibitory concentration (MIC) determined by the early time point incubation falls within ± one twofold dilution of CI. Categorical agreement (CA) was calculated as the percentage of isolates for which the interpretive category (susceptible, intermediate, or resistant) is the same between MLA cultures and CI cultures. Very major error (VME) is defined as isolates classified as resistant with the CI culture but classified as susceptible with the MLA culture. Major error (ME) is defined as isolates classified as susceptible by the CI culture but resistant by the MLA culture. Minor error (mE) is defined as one method yielding an intermediate category while the other yielded either susceptible or resistant result. Discrepancies were analyzed by drug and organism group. EA, CA, VME, ME, and mE were calculated in accordance with CLSI guidelines. Acceptability thresholds were applied as follows: EA ≥ 90%, CA ≥ 90%, VME ≤ 1.5%, ME ≤ 3%, and mE ≤ 10%.

## RESULTS

### Optimization of culture incubation durations on MLA by specimen type

#### Sterile sources

**Fig 1:**
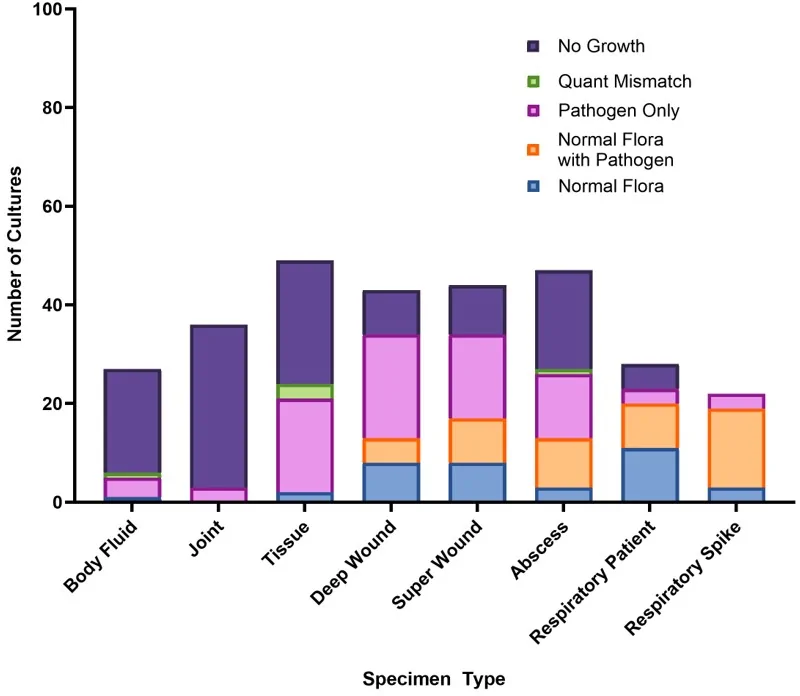
Quantitation across specimen types. Quantitation values for specimen types (body fluid, joint, tissue, deep wound, superficial wound, abscess, respiratory patient, and respiratory spike) had the following results: no growth, normal flora, normal flora with pathogen, and pathogen-only growth. Quantitative mismatches between methods were infrequent and are summarized by specimen source.

A total of 110 specimens (46 tissues, 37 joints, and 27 body fluids) were set up using both MLA and CI as described in the methods. Of the 110 cultures analyzed, 25 were positive for clinically significant pathogens. The positive cultures were RTR on MLA at the following times: 1 of 25 (4%) at 10 h, 5 of 25 (20%) at 12 h, and 9 of 25 (36%) at 14 h, 15 of 25 (60%) by 16 h, 21 of 25 (84%) at 18 h, 23 of 25 (92%) at 24 h, and 25 of 25 (100%) at 36 h (Table 1). No additional significant growth was observed between the 36 h and 72 h time points. The two cultures, one from a maxillary sinus and the other from amniotic fluid, that reached RTR only at 36 h were suspected colonies of aerotolerant anaerobic *Actinomyces* spp., which were ultimately considered clinically insignificant and were observed on CI. Additionally, the routine aerobic clinical cultures also did not report growth of *Actinomyces* spp. in these two specimens. Using CI, four additional specimens grew a single colony of bacteria on a single medium and were determined to be contaminants, as such growth was not reported from routine clinical cultures and was not used in the accuracy calculations. Three specimens demonstrated discordant results between the MLA and CI. In one case, rare growth of *Enterococcus* spp. was observed on CI, while no growth was identified on the MLA. On investigation, the routine clinical culture with the same specimen was reported as no growth. The inconsistent culture results from the same specimen using different culture settings suggest either sample variability, low colony-forming unit (CFU/mL) count, or possible contamination. In another case, *Pseudomonas aeruginosa* was detected on the MLA culture but not on the CI. However, *P. aeruginosa* was also isolated from routine clinical culture, supporting the MLA result and indicating a possible false negative with CI culture. In the third discordant culture, rare growth of *Candida tropicalis* was identified from CI, which was not detected by MLA. The routine clinical culture also reported *C. tropicalis*, suggesting that the discordant result may have stemmed from low fungal load or plate-specific variability. There were four instances of differences in the amount of growth seen between the MLA and CI cultures; in all cases, the MLA had more growth than the CI cultures (Fig. 1). Overall, the accuracy of MLA was 97.3% compared to CI, demonstrating strong concordance and reliability for the early detection of clinically relevant pathogens from MLA (Table 2).

**TABLE 2:** Normal flora and pathogen comparison per source

Source	No growth	Normal flora	Normal flora with pathogen	Pathogen only	Quantitation discordant	Accuracy of MLA vs CI
Sterile sources	79	3	0	25	4	97.3%
Deep wound	9	8	5	21	0	93.0%
Super wound	10	8	9	17	0	95.5%
Abscess	15	4	3	25	1	95.7%
Respiratory	5	11	9	3	0	100.0%
Respiratory contrived	0	3	16	3	0	100.0%
Urine^*a*^	46	3	7	48	7	95.2%

^*a*^ No growth includes <10 CFU/mL insignificant growth and contaminant.

#### Abscesses

A total of 47 specimens were set up using MLA and CI. The MLA yielded 30 positive cultures that were RTR at the following times: 0 of 30 (0%) at 10 h, 0 of 30 (0%) at 12 h, 8 of 30 (27%) at 14 h, 15 of 30 (50%) at 16 h, 22 of 30 (73%) at 18 h, 27 of 30 (90%) at 24 h, and 30 of 30 (100%) at 36 h (Table 1). No additional clinically significant organisms were recovered with incubation beyond 36 h. All pathogens recovered by CI were recovered by MLA at earlier time points. Two specimens yielded additional organisms on MLA. One sample grew a viridans streptococcus in addition to the *S. aureus* that was also observed from CI. However, the viridans streptococcus was not reported from routine clinical culture. In another specimen, a single colony of *Enterobacter cloacae* was detected using MLA, while no growth was observed with CI. When the specimen was processed for routine clinical culture, it yielded light growth of *E. cloacae*. There was one occurrence of quantitation differences between the CI and MLA, with a single MLA culture growing more than the CI culture (Fig. 1). The accuracy of MLA compared to CI was 95.7% due to two additional organisms isolated in two specimens that were not isolated from CI (Table 2).

#### Deep wounds

A total of 43 specimens were set up using MLA and CI. The MLA yielded 25 positive cultures that were RTR at the following times: 0 of 25 (0%) at 10 h, 1 of 25 (4%) at 12 h, 7 of 25 (28%) at 14 h, 13 of 25 (52%) at 16 h, 19 of 25 (76%) at 18 h, and 25 of 25 (100%) at 24 h (Table 1). No additional significant aerobic organisms were observed with incubation beyond 36 h. However, one specimen showed breakthrough growth of *Cutibacterium avidum* at 72 h of incubation. The MLA successfully isolated all aerobic pathogens observed in CI, while it also yielded additional growth in five specimens not observed from CI. In two specimens, MLA yielded pathogens originally reported in routine clinical culture (one *Pasteurella multocida* and one *Staphylococcus lugdunensis*). In another two specimens, MLA yielded rare normal skin flora and a single colony of coagulase-negative staphylococcus, respectively. Routine clinical cultures reported light growth of normal skin flora for these specimens. For the remaining specimen, MLA yielded the primary pathogen (*Proteus* spp.) that was also isolated from CI, while additional beta-hemolytic colonies were observed from MLA plates (Fig. 1). The accuracy of MLA compared to CI was 93.0% (Table 2). The two specimens growing only rare skin flora and anaerobic breakthrough of *C. avidum* were excluded from the calculation.

#### Superficial wounds

A total of 44 specimens from superficial wounds were set up in parallel using MLA and CI. The MLA resulted in 22 positive cultures that were RTR at the following time points: 0 of 22 (0%) at 10 h, 0 of 22 (0%) at 12 h, 2 of 22 (9%) at 14 h, 13 of 22 (59%) at 16 h, 18 of 22 (82%) at 18 h, 19 of 22 (86%) at 20 h, 19 of 22 (86%) at 22 h, and 22 of 22 (100%) at 24 h (Table 1). No additional growth was observed at the 36 h and 72 h time points. No significant difference in semi-quantitative growth of the pathogens and/or normal flora was observed between MLA and CI (Table 2). Differences in results between MLA and CI were observed in three specimens. For the first specimen, MLA yielded small pinpoint colonies identified as *Streptococcus pyogenes* and *S. aureus*, which were absent from CI. The growth of *S. pyogenes* and *S. aureus* was also reported from routine clinical culture. For the second specimen, MLA yielded light growth of *S. marcescens* on the chocolate plate only, which was absent from CI. However, the organism was also reported from routine clinical culture. The last specimen grew rare skin flora from MLA, with no growth from CI. The routine clinical culture reported light growth of skin flora (Fig. 1). The accuracy of MLA was 95.5% due to additional organisms being recovered from MLA (Table 2). The culture growing light amounts of skin flora was excluded from the calculation.

#### Respiratory specimens

A total of 50 respiratory specimens were set up using MLA and CI, including 28 patient specimens and 22 spiked specimens (residual respiratory specimens inoculated with common respiratory pathogens; see Table S2). The MLA yielded 29 positive cultures that were RTR at the following time points: 0 of 29 (0%) at 10 h, 3 of 29 (10%) at 12 h, 13 of 29 (45%) at 14 h, 22 of 29 (76%) at 16 h, 26 of 29 (90%) at 18 h, 28 of 29 (97%) at 24 h, and 29 of 29 (100%) at 36 h. No additional pathogens were observed after 36 h of incubation using MLA (Table 2). All specimens tested yielded matching results using the MLA and CI (Fig. 1). Interestingly, five spiked specimens (one *S. pneumoniae*, two *Haemophilus influenzae*, and two *Haemophilus parainfluenzae*) only yielded normal flora using both culture systems (Table 2). The accuracy of MLA was 100% when compared to CI.

#### Urine specimens

A total of 104 urine specimens were set up using MLA and CI. The MLA yielded 55 cultures positive for a uropathogen that were RTR at the following time points: 4 of 55 (7%) cultures at 12 h, 45 of 55 (82%) at 14 h, 50 of 55 (91%) at 16 h, and 55 of 55 (100%) at 18 h (Table 2). All five pathogens RTR at 18 h were also observed at the 16 h time point. However, at the 16 h time point, there were insufficient isolated colonies or cell mass to perform both MALDI-TOF MS identification and AST. Of the 104 specimens evaluated, 9 demonstrated discrepant results between the MLA and CI. For five specimens, MLA yielded insignificant growth (<10,000 CFU/mL) or normal urogenital flora, while CI yielded no growth. These differences between MLA and CI are clinically insignificant, as both insignificant growth and normal urogenital flora would not be worked up according to laboratory protocol. These five discrepant results were not counted as discrepant in the calculation of the accuracy. Two urine specimens isolated a significant urogenital pathogen (>100 K CFU/mL *Escherichia coli*) from CI, while the MLA yielded >100 K CFU/mL *E. coli* with the addition of <10 K CFU/mL of insignificant growth. Another specimen observed >100 K CFU/mL *Proteus mirabilis* and <10 K CFU/mL of insignificant growth by the CI, while the MLA reported >100 K *P*. *mirabilis* and 25 K–50 K CFU/mL of *Lactobacillus* spp., which is considered normal genital flora. The last discrepant result showed <10K insignificant growth and a single colony indicative of *Streptococcus agalactiae* from CI, while 10 K-25 K CFU/mL of *S. agalactiae* were isolated from MLA (Fig. 1). The accuracy of MLA was 95.2%, which was due to additional organisms reported from MLA in five urine specimens. Among 55 positive cultures, quantitative results revealed that seven specimens yielded greater growth using MLA compared to CI. The quantitation accuracy for MLA was 93.3% when compared to CI (Table 2). Of note, minimal changes were observed in urine culture quantitation between the 12 h and 18 h time points; these differences had no impact on the reporting of pathogenic organisms according to the laboratory’s procedure.

### MALDI-TOF MS identification at early time points from SI cultures

**Fig 2:**
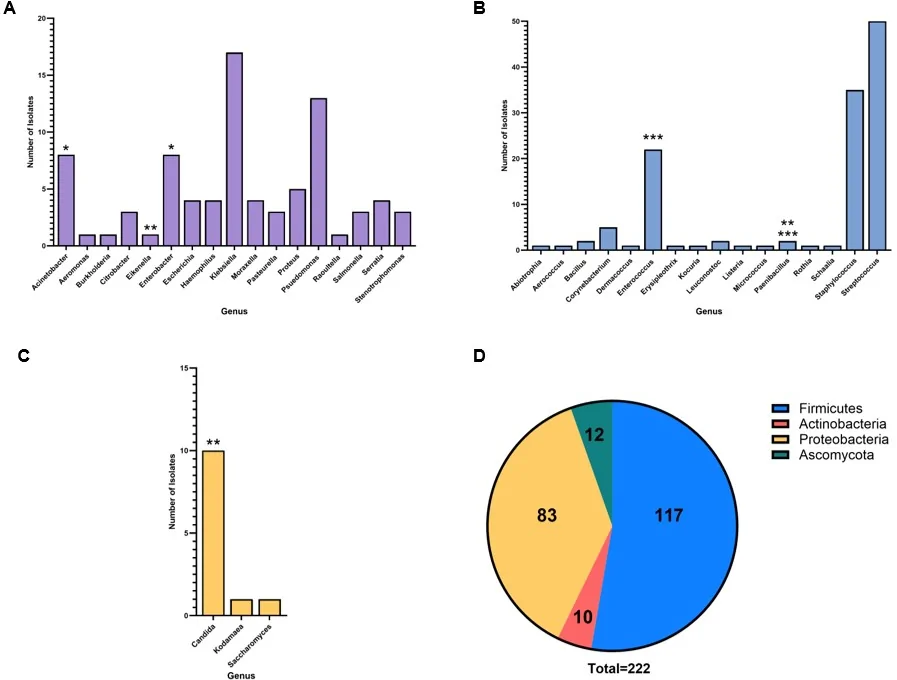
MALDI-TOF identification after WASPLab early incubation. (**A**) Genus of gram-negative organisms used on MALDI-TOF for identification at 10 h and 16 h. (**B**) Genus of gram-positive organisms used on MALDI-TOF for identification. (**C**) Genus of yeasts used on MALDI-TOF for identification. (**D**) Total number of organisms organized by phyla used in the MALDI-TOF study. Identification was successful for all listed species except for *Paenibacillus*, *Enterococcus dispar*, *Acinetobacter radioresistens*, *Eikenella corrodens*, *Enterobacter asburiae*, and *Candida lusitaniae*. Symbols indicate identification outcomes: *, identified at 10 h only; **, identified at 16 h only; ***, not identified at either time point.

To evaluate the accuracy of MALDI-TOF MS at early time points, identification results from MLA culture colonies at 10 h and 16 h were compared to results from CI cultures with at least 18 h of incubation. A total of 222 isolates, including 127 gram-positive bacteria, 83 gram-negative bacteria, and 12 yeast isolates were tested (Fig. 2). All organisms included in this study are detailed in Table S1.

Out of the 83 gram-negative organisms tested, 82 (98.8%) were accurately identified by MALDI-TOF MS at the 10 h time point, while 81 (97.6%) were accurately identified at the 16 h time point (Fig. 2A). Specifically, one *Eikenella corrodens* isolate failed to yield an identification at the 10 h time point, while one *Acinetobacter radioresistens* isolate and one *Enterobacter asburiae* isolate were not identified at the 16 h time point. The accuracy of MALDI-TOF MS for gram-positive organisms was 97.6% at the 10 h time point and 98.4% at the 16 h time point, respectively, when compared to identification results from CI cultures (Fig. 2B). One *Paenibacillus* spp. isolate failed to yield identification at the 10 h time point, while a different *Paenibacillus* isolate failed to yield an identification at both the 10 h and 16 h time points. Additionally, one *Enterococcus dispar* isolate did not produce identification at either time point. Regarding the yeast isolates, 11 of the 12 organisms were accurately identified at the 10 h and 12 of 12 were identified at the 16 h time points. An isolate of *Candida lusitaniae* was only identified at the 16 h time point. The accuracy of identification for yeast was 91.6% at the 10 h time point and 100% at the 16 h time point (Fig. 2C).

### AST at early incubation time points from MLA cultures

#### AST for gram-negative organisms using Vitek 2 GN-807N card

A total of 50 isolates, including 35 Enterobacterales, 8 *P. aeruginosa,* and 7 *Acinetobacter* spp., were tested after 10 h and 16 h of incubation in the MLA. Among these isolates, 13 were carbapenem-resistant organisms, and seven were ESBL-producing organisms. The AST results were compared to those obtained after the standard incubation time of 18–24 h in conventional incubators prior to AST testing. The overall AST results obtained from colonies with 10 h and 16 h incubation durations in MLA were comparable to those obtained from colonies incubated for 18-24 h in CI. EA was greater than 93% for all antimicrobials in the panel tested at the 10 h time point and greater than 96% for all antimicrobials tested at the 16 h time point. Categorical agreement also showed strong concordance at both the 10 h and 16 h time points. All antimicrobial/organism combinations were acceptable apart from four antimicrobials: non-urine breakpoint for cefazolin, ceftriaxone, piperacillin/tazobactam, and nitrofurantoin (Table 3). The cefazolin non-urine breakpoint resulted in 1 ME at both the 10 h and 16 h time points, resulting in a 50% ME rate for both time points. Such a high ME rate was due to the low number of susceptible isolates tested in the study (*N* = 2). Ceftriaxone produced a single major error at the 10 h time point yielding a 4% major error rate. Piperacillin/tazobactam at the 10 h time point produced 1 VME for a *P. aeruginosa* isolate, resulting in a 7.7% VME rate. Nitrofurantoin produced five mE resulting in a 15% mE rate at both the 10 h and 16 h time points, and one VME at the 16 h time point, resulting in a 7% VME rate.

**TABLE 3:** AST results for gram-negative bacteria using at MLA incubations of 10 h and 16 h compared to CI routine incubation time

Antibiotics	No. of results	10 h							16 h						
		mE (*n*)	% mE	ME (*n*)	% ME	VME (*n*)	% VME	% EA	mE (*n*)	% mE	ME (*n*)	% ME	VME (*n*)	% VME	% EA
Ampicillin	35	0	0	0	0	0	0	100	0	0	0	0	0	0	100
Ampicillin/sulbactam	42	3	7.1	0	0	0	0	100	3	7.1	0	0	0	0	100
Cefazolin (non-urine)	33	1	3.0	1	50	0	0	96.7	2	6.1	1	50	0	0	97.0
Cefazolin (urine)	33	0	0	0	0	0	0	97	0	0	0	0	0	0	97
Cefuroxime	32	2	6.3	0	0	0	0	97	0	0	0	0	0	0	100
Cefepime	40	2	5	0	0	0	0	95	2	5	0	0	0	0	97
Ceftazidime	47	2	4.3	0	0	0	0	98	3	6.4	0	0	0	0	98
Ceftriaxone	40	0	0	1	4	0	0	97	0	0	0	0	0	0	100
Ertapenem	35	0	0	0	0	0	0	97	2	5.7	0	0	0	0	100
Meropenem	46	2	4.3	0	0	0	0	100	1	2.2	0	0	0	0	100
Gentamicin	41	0	0	0	0	0	0	100	0	0	0	0	0	0	100
Ciprofloxacin	50	1	2	0	0	0	0	98	1	2	0	0	0	0	96
Levofloxacin	50	1	2	0	0	0	0	100	0	0	0	0	0	0	100
Piperacillin/tazobactam	46	3	6.5	0	0	1	7.7	93	2	2.2	0	0	0	0	96
Nitrofurantoin	34	5	15	0	0	0	0	97.1	5	15	0	0	1	7	97.1
Trimethoprim/sulfamethoxazole	29	0	0	0	0	0	0	100	0	0	0	0	0	0	100

#### AST for gram-positive organisms using Vitek 2 AST-GP75 card

A total of 49 isolates were tested, comprising 20 *Enterococcus* spp., 18 *S. aureus*, and 11 coagulase-negative staphylococci. The AST results from isolates incubated for 10 h and 16 h in MLA were compared to those obtained after standard incubation duration (18–24 h) in CI. All antimicrobials tested at both the 10 h and 16 h time points were within the acceptability range for VME, ME, and mE when compared to standard incubation time in CI except for doxycycline at the 10 h time point (Table 4). The mE rate for doxycycline at the 10 h time point was 10.2%, slightly exceeding the acceptable range. Additionally, the study found only a single EA error for oxacillin in a *S. aureus* isolate, resulting in 97% EA agreement. All other antibiotics tested in this group had 100% EA.

**TABLE 4:** AST results for gram-positive bacteria at MLA incubations of 10 h and 16 h compared to CI routine incubation time

Antibiotics	No. of results	10 h							16 h						
		mE (*n*)	% mE	ME (*n*)	% ME	VME (*n*)	% VME	% EA	mE (*n*)	% mE	ME (*n*)	% ME	VME (*n*)	% VME	% EA
Ampicillin	20	0	0	0	0	0	0	100	0	0	0	0	0	0	100
Cefoxitin	29	0	0	0	0	0	0	100	0	0	0	0	0	0	100
Ciprofloxacin	49	0	0	0	0	0	0	100	0	0	0	0	0	0	100
Clindamycin	29	0	0	0	0	0	0	100	0	0	0	0	0	0	100
Daptomycin	42	1	2.4	0	0	0	0	100	2	5	0	0	0	0	100
Doxycycline	49	5	10.2	0	0	0	0	100	3	6.1	0	0	0	0	100
Erythromycin	49	0	0	0	0	0	0	100	1	2	0	0	0	0	100
Gentamicin	29	1	3.4	0	0	0	0	100	0	0	0	0	0	0	100
Levofloxacin	49	0	0	0	0	0	0	100	0	0	0	0	0	0	100
Linezolid	49	1	2.0	0	0	0	0	100	0	0	0	0	0	0	100
Moxifloxacin	29	2	6.9	0	0	0	0	100	2	6.9	0	0	0	0	100
Nitrofurantoin	49	1	2	0	0	0	0	100	1	2	0	0	0	0	100
Oxacillin	29	0	0	0	0	0	0	100	0	0	0	0	0	0	97
Rifampin	29	0	0	0	0	0	0	100	0	0	0	0	0	0	100
Tigecycline	49	0	0	0	0	0	0	100	0	0	0	0	0	0	100
Trimethoprim/sulfamethoxazole	29	0	0	0	0	0	0	100	0	0	0	0	0	0	100
Vancomycin	49	0	0	0	0	0	0	100	0	0	0	0	0	0	100

#### AST for *Streptococcus* spp. using Vitek 2 AST-ST02 card

A total of 52 isolates were tested after 10 h and 16 h of incubation in the MLA, and the results were compared to those obtained after standard incubation time (18–24 h) in CI. The study evaluated AST for 13 β-hemolytic streptococci, 14 *S. anginosus* group, 5 *S. dysgalactiae* complex, 1 *S. equinus* group, 15 *S. mitis* group (of which 9 were *S. pneumoniae*)*,* and 4 *S. salivarius* group. EA was greater than 91% for all antimicrobials in the panel tested at the 10 h time point and greater than 93% tested at the 16 h time point (Table 5). All antimicrobials tested were within the acceptability range for VME, ME, and mE, except for ceftriaxone (when applying the *S. pneumoniae* meningitis breakpoints), linezolid, and moxifloxacin. A single incidence of mE for ceftriaxone against *S. pneumoniae* (using meningitis breakpoints) was identified in this evaluation, resulting in an mE rate of 11.1%. This slightly elevated mE rate was due to the limited number of *S. pneumoniae* isolates included in the study (*N* = 9). Three VMEs were identified for linezolid at the 10 h time point, resulting in a VME rate of 6.5%. Notably, all the VMEs were associated with *S. anginosus* isolates. At the 16 h time point, the ME rate for linezolid and moxifloxacin was 4.3% and 6.7%, respectively. Both exceeded the acceptability ME range.

**TABLE 5:** AST results for *Streptococcus* species at MLA incubations of 10 h and 16 h compared to CI routine incubation time

Antibiotics	No. of results	10 h							16 h						
		mE (*n*)	% mE	ME (*n*)	% ME	VME (*n*)	% VME	% EA	mE (*n*)	% mE	ME (*n*)	% ME	VME (*n*)	% VME	% EA
Ampicillin	35	1	2.9	0	0	0	0	100	1	2.9	0	0	0	0	100
Penicillin	41	1	2.4	0	0	0	0	100	1	2.4	0	0	0	0	100
Penicillin (men)	9	0	0	0	0	0	0	100	0	0	0	0	0	0	100
Penicillin (non-men)	9	0	0	0	0	0	0	100	0	0	0	0	0	0	100
Cefotaxime	40	0	0	0	0	0	0	100	0	0	0	0	0	0	100
Ceftriaxone	41	1	2.4	0	0	0	0	97.6	1	2.4	0	0	0	0	97.6
Ceftriaxone (men)	9	0	0	0	0	0	0	100	1	11.1	0	0	0	0	100
Ceftriaxone (non-men)	9	0	0	0	0	0	0	100	0	0	0	0	0	0	100
Clindamycin	41	1	2.4	0	0	0	0	100	1	2.4	0	0	0	0	100
Erythromycin	42	0	0	0	0	0	0	100	0	0	0	0	0	0	100
Levofloxacin	48	0	0	0	0	0	0	100	0	0	0	0	0	0	100
Linezolid	46	0	0	1	2.2	3	6.5	91.3	0	0	2	4.4	0	0	95.7
Moxifloxacin	45	0	0	0	0	0	0	100	2	4.4	3	6.7	0	0	93.3
Tigecycline	46	0	0	0	0	0	0	100	0	0	0	0	0	0	100
Vancomycin	47	0	0	0	0	0	0	93.6	0	0	0	0	0	0	93.6

## DISCUSSION

Optimizing imaging times is critical when incorporating MLA systems into the clinical microbiology workflow. The laboratory not only needs to optimize the flow of plates into the MLA system but also must account for the effects of MLA incubation on downstream applications such as identification and susceptibility testing. Excessive image acquisition can cause an imaging backlog, thus delaying image acquisition for all cultures incubated within the MLA. If plates are read without enough cellular mass or too early for reliable identification and/or susceptibility testing, then earlier reading is less efficient because additional incubation is necessary to complete the culture workup. Our study sought to optimize MLA workflow, allowing earlier plate reading times while having minimal effects on the reliability of downstream testing for identification and susceptibility testing.

**Fig 3:**
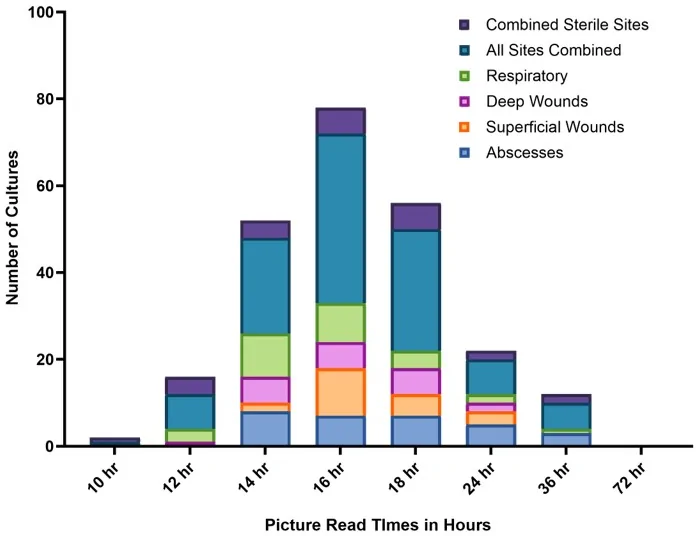
Optimization of MLA culture workup times from all sources (excluding urine specimens). Within this group, there were 110 specimens that had image read times of 10, 12, 14, 16, 18, 24, 36, and 72 h. Each time point was evaluated to determine if the culture was ready to be worked up, meaning visual inspection of the culture image to ascertain if colony growth was sufficient so that it could be used for identification and AST studies.

The study aimed to identify the optimal plate reading time points by determining the earliest time point at which 50% of cultures were ready to work up, an intermediate time point at which 75% of cultures were ready to work up, and the latest time point at which no additional growth of clinically significant aerobic pathogens was observed. Our data found that greater than 50% of positive cultures from all tested sources are RTR at 16 h of incubation in the MLA, and no significant aerobic pathogens appeared after an incubation duration of 36 h (Fig. 3). Of note, one culture from a sterile source grew *C. tropicalis* in the CI culture but not in the MLA culture. However, the *C. tropicalis* also failed to grow in the MLA culture after prolonged incubation duration (72 h), suggesting that the discrepancy was most likely due to a low organism load in the specimen and represents a false negative result. Additionally, our study revealed minimal changes in growth quantitation of either pathogenic organisms or flora after 16 h of incubation, allowing predominating organisms to be evaluated at early incubation time points (Fig. 3). The study found that 5 out of 10 discrepant results from non-urine specimens were attributed to the growth of additional organisms in the MLA culture compared to CI culture. These organisms were pathogens that had been previously reported in routine clinical cultures. These findings suggest that incubation in MLA may enhance the recovery of organisms present in low quantities. Interestingly, the MLA cultures tended to have fewer one-colony contaminants on the plates, which may be due to less manipulation of the sample during setup and because the WASP is a closed system. Based on these results, the optimal earliest time point for imaging and plate reading was set at 16 h after incubation, the intermediate time point was set at 24 h, and the final time point was determined to be 36 h. For urine cultures, a single imaging time point at 16 h was chosen, as more than 90% of cultures were RTR and all pathogens were present at this time point. However, a few slowly growing organisms did not have sufficient cell mass for further workup and required additional incubation before identification and AST could be performed.

The study also demonstrated that MALDI-TOF MS identification is minimally affected using colonies at earlier incubation time points in MLA cultures. All isolates had sufficient growth to prepare the MALDI-TOF MS slide at the 10 h and 16 h time points. Overall, the early time points had almost equivalent performance when compared to CI, with 97.7% and 98.2% agreement at the 10 h and 16 h time points, respectively. All discrepancies in this study were attributed to a “no identification” result at the earlier time points, with none resulting in a misidentification. Although a smaller quantity of yeasts was included in the study, each species was appropriately identified at earlier time points. These data support that MALDI-TOF MS is reliable in identification at early time points using MLA. Some species may require additional incubation for reliable species-level identification when implementing early MLA identification workflows in clinical laboratories, particularly for organisms with slower growth kinetics or unique phenotypic characteristics. While the study did not include an exhaustive list of all bacteria, it did contain the most common species, including some fastidious organisms encountered in a clinical microbiology laboratory (Fig. 2).

Recently, Osborn et al. reported that early growth isolates incubated for as short as 6 h in conventional incubators and tested by BMD and the automated Phoenix system gave equivalent AST results compared to standard incubation time (35). Our study found a similar trend, showing high concordance rates of AST results using cultures grown at the 10 h and 16 h time points in MLA compared to standard incubation time in CI, with >90% essential agreement with all drugs tested with the Vitek 2 AST system (36).

While the study provides preliminary evidence suggesting that incubation time in MLA can be reduced with minimal impact on MALDI-TOF MS identifications and AST, it is important to acknowledge that our study has certain limitations. For early time points AST evaluation, the isolates tested were skewed toward resistant phenotypes. Including more susceptible strains would offer a more balanced and comprehensive assessment of AST performance using cultures at early time points. Furthermore, the robustness of AST evaluation could be strengthened by including additional isolates from key species, such as *S. pneumoniae* and *P. aeruginosa*. Additionally, the high mE rate for nitrofurantoin may be attributed to the substantial proportion (22%) of *Klebsiella oxytoca* and *Klebsiella pneumoniae* isolates included in the evaluation, which have been reported to have an MIC_50_ of 64 µg/mL that bisects the breakpoint (37). Another key limitation of this study is that earlier culture read times were evaluated outside the manufacturer-specified incubation window for CHROMagar Orientation (18–24 h), and therefore represent off-label use; results at reduced time points should be interpreted with caution until supported by further validation.

The use of MLA systems allows earlier initial reads and the ability to finalize cultures 12 to 36 h earlier compared to standard manual laboratory procedures. As suggested by previous studies, the impact on turnaround times for earlier culture reads varies between laboratories according to staffing models (38). The impact may be minimal if plates are read on a single shift, while the impact could be significant if plates are read across multiple shifts. According to the workflow in our laboratory, which utilizes MLA and incorporates earlier plate reading and AST workup across multiple shifts, some AST results for positive cultures are available within 30 h of plating. With MLA, the ability to shorten incubation times not only increases specimen throughput but also enhances overall efficiency. The reduction in total incubation times enabled by MLA supports faster turnaround times for reporting cultures with complete organism identification and susceptibility profiles. This, in turn, has the potential to provide more rapid, actionable results that can positively impact patient care.

### Conclusion

In this study, MLA incubation and image acquisition times were optimized for the most common culture types encountered in the clinical microbiology laboratory using the Copan WASPLab system. The findings demonstrated that cultures can be initially read at 16 h, with a final read at 36 h, with equivalent accuracy to cultures initially read at 18–24 h and finalized at up to 72 h using conventional culture systems. Furthermore, the study provided data indicating minimal impact on the accuracy of organism identification and AST results using the Vitek Prime MS systems and Vitek 2 AST with cultures obtained at early time points in MLA.

## SUPPLEMENTAL MATERIAL

Table S1 jcm.00232-26-s0001.docx Organism list for MALDI-TOF MS identification.

Table S2 jcm.00232-26-s0002.docx Contrived respiratory samples used in optimization studies.

Table S3 jcm.00232-26-s0003.docx Discordant AST results for Gram-positives bacteria.

Table S4 jcm.00232-26-s0004.docx Discordant AST results for *Streptococcus* species.

Table S5 jcm.00232-26-s0005.docx Discordant AST results for Gram-negative bacteria.

ASM does not own the copyrights to Supplemental Material that may be linked to, or accessed through, an article. The authors have granted ASM a non-exclusive, world-wide license to publish the Supplemental Material files. Please contact the corresponding author directly for reuse.
