# Supplementary material for: Optimization of culture read times using full microbiology laboratory automation
Source: J Clin Microbiol. 2026 Sep 18;64(10):e00232-26. doi: 10.1128/jcm.00232-26 (PMC13712942; doi:10.1128/jcm.00232-26)
Supplement: Supplementary file 1 [file jcm.00232-26-s0001.docx]

**Supplemental Table 1. Organisms list for MALDI- TOF MS identification study**

| **Organism** | **Number of Isolates** | **Organism** | **Number of Isolates** |
| --- | --- | --- | --- |
| *Abiotrophia defectiva* | 1 | *Micrococcus letus* | 1 |
| *Acinetobacter baumannii* | 5 | *Moraxella catarrhalis* | 1 |
| *Acinetobacter baumannii complex* | 1 | *Morganella morganii* | 3 |
| *Acinetobacter pittii* | 1 | *Paenibacillus species* | 2 |
| *Acinetobacter radioresistens* | 1 | *Pastuerella multocida* | 2 |
| *Aerococcus viridans* | 1 | *Pasturella canis* | 1 |
| *Aeromonas veronii* | 1 | *Proteus mirabilis* | 5 |
| *Bacillus cereus* | 2 | *Psuedomonas aeruginosa* | 10 |
| *Burkholderia cepacia complex* | 1 | *Psuedomonas oryzihibitans* | 1 |
| *Candida albicans* | 1 | *Psuedomonas putida* | 2 |
| *Candida duobushaemulonis* | 1 | *Raoultella orinthinolytica* | 1 |
| *Candida haemuloni* | 1 | *Rothia mucilaginosa* | 1 |
| *Candida keyfr* | 1 | *Saccharomyces cerevisiae* | 1 |
| *Candida krusei* | 1 | *Salmonella enterica* | 1 |
| *Candida lusitaniae* | 1 | *Salmonella enterica ssp enterica* | 2 |
| *Candida parapsilosis* | 2 | *Schaalia odontolyticus* | 1 |
| *Candida pelliculosa* | 1 | *Serratia marscens* | 4 |
| *Candida tropacalis* | 1 | *Staphylococcus aureus* | 20 |
| *Citrobacter braakii* | 1 | *Staphylococcus capitis* | 2 |
| *Citrobacter freundii* | 2 | *Staphylococcus epidermidis* | 6 |
| *Corynebacterium amycolatum* | 2 | *Staphylococcus haemolyticus* | 2 |
| *Corynebacterium striatum* | 3 | *Staphylococcus hominis* | 2 |
| *Dermacoccus spp.* | 1 | *Staphylococcus lugdunensis* | 1 |
| *Eikenella corrodens* | 1 | *Staphylococcus simulans* | 2 |
| *Enterobacter asburiae* | 1 | *Stenotrophomonas maltophilia* | 3 |
| *Enterobacter cloacae complex* | 7 | *Streptococcus agalactiae* | 9 |
| *Enterococcus dispar* | 1 | *Streptococcus anginosus* | 5 |
| *Enterococcus faecalis* | 14 | *Streptococcus constellatus* | 2 |
| *Enterococcus faecium* | 7 | *Streptococcus dysgalactiae* | 3 |
| *Erysipleothrix species* | 1 | *Streptococcus intermedius* | 3 |
| *Escherichia coli* | 4 | *Streptococcus lutetiensis* | 1 |
| *Haemophilus influenzae* | 3 | *Streptococcus mitis/oralis group* | 7 |
| *Klebsiella aerogenes* | 3 | *Streptococcus pneumoniae* | 10 |
| *Klebsiella oxytoca* | 6 | *Streptococcus pyogenes* | 6 |
| *Klebsiella pneumoniae* | 8 | *Streptococcus salivarius* | 1 |
| *Kocuria kristinae* | 1 | *Streptococcus salivarius ssp. salivarius* | 1 |
| *Kodamaea ohmeri* | 1 | *Streptococcus salivarius/vestibularis* | 1 |
| *Leuconostoc mesenteroides* | 2 | *Streptococcus thermophilus* | 1 |
| *Listeria monocytogenes* | 1 |  |  |

**Supplemental Table 1.** Comprehensive list of organisms and number of isolates included in the MALDI-TOF MS identification study.
