# Supplementary material for: Optimization of culture read times using full microbiology laboratory automation
Source: J Clin Microbiol. 2026 Sep 18;64(10):e00232-26. doi: 10.1128/jcm.00232-26 (PMC13712942; doi:10.1128/jcm.00232-26)
Supplement: Supplementary file 2 [file jcm.00232-26-s0002.docx]

**Supplemental Table 2. Contrived Respiratory Samples used in Optimization Studies**

| **Specimen Number** | **Spiked Organism** |
| --- | --- |
| RESP 1 | *Stenotrophomonas maltophilia* |
| RESP 2 | *Haemophilus influenzae* |
| RESP 3 | *Pseudomonas aeruginosa* 9721 |
| RESP 4 | *Streptococcus pneumoniae* 9464-14678 |
| RESP 5 | *Elizabethkingia meningoseptica* |
| RESP 6 | *Pseudomonas aeruginosa*  BAA-1744 |
| RESP 7 | *Streptococcus pneumoniae* |
| RESP 8 | *Haemophilus influenzae* |
| RESP 9 | *Haemophilus parainfluenzae* |
| RESP 10 | *Haemophilus parainfluenzae* |
| RESP 11 | *Stenotrophomonas maltophilia* |
| RESP 12 | *Haemophilus influenzae* |
| RESP 13 | *Pseudomonas aeruginosa* 9721 |
| RESP 14 | *Streptococcus pneumoniae* 9464-14678 |
| RESP 15 | *Elizabethkingia meningoseptica* |
| RESP 16 | *Pseudomonas aeruginosa*  BAA-1744 |
| RESP 17 | *Streptococcus pneumoniae* |
| RESP 18 | *Haemophilus influenzae* |
| RESP 19 | *Haemophilus parainfluenzae* |
| RESP 20 | *Haemophilus parainfluenzae* |
| RESP 21 | *Stenotrophomonas maltophilia* |
| RESP 22 | *Haemophilus influenzae* |

**Supplemental Table 2.** Composition of contrived respiratory specimens used for optimization studies, including specimen identifiers and spiked organisms
