# Supplementary material for: Optimization of culture read times using full microbiology laboratory automation
Source: J Clin Microbiol. 2026 Sep 18;64(10):e00232-26. doi: 10.1128/jcm.00232-26 (PMC13712942; doi:10.1128/jcm.00232-26)
Supplement: Supplementary file 3 [file jcm.00232-26-s0003.docx]

**Supplemental Table 3. Discordant AST Results for Gram-positives Bacteria at MLA Incubations of 10 h and 16 h Compared CI Routine Incubation Time**

| **Antibiotics** | ***E. dispar*** | | | ***E. faecalis*** | | | ***E. faecium*** | | | ***S. aureus*** | | |
| --- | --- | --- | --- | --- | --- | --- | --- | --- | --- | --- | --- | --- |
|  | **CI** | **10 h** | **16 h** | **CI** | **10 h** | **16 h** | **CI** | **10 h** | **16 h** | **CI** | **10 h** | **16 h** |
| **Daptomycin** |  |  |  | I, I | S, I | S, S |  |  |  |  |  |  |
| **Doxycycline** |  |  |  | I, R, I, I, R | I, I, S, R, I | R, I, I, R, R |  |  |  |  |  |  |
| **Gentamicin** |  |  |  |  |  |  |  |  |  | I | R | I |
| **Linezolid** | S | I | I |  |  |  |  |  |  |  |  |  |
| **Moxifloxacin** |  |  |  |  |  |  |  |  |  | I | R | I |
| **Nitrofurantoin** |  |  |  | S | S | I | I | S | I |  |  |  |

**Supplemental Table 3.** Discordant antimicrobial susceptibility testing (AST) results for Gram-positive bacteria comparing MLA incubation at 10 h and 16 h versus conventional incubation (CI).
