# Supplementary material for: Optimization of culture read times using full microbiology laboratory automation
Source: J Clin Microbiol. 2026 Sep 18;64(10):e00232-26. doi: 10.1128/jcm.00232-26 (PMC13712942; doi:10.1128/jcm.00232-26)
Supplement: Supplementary file 4 [file jcm.00232-26-s0004.docx]

**Supplemental Table 4. Discordant AST results for *Streptococcus* species at MLA Incubations of 10 h and 16 h Compared CI Routine Incubation Time**

| **Antibiotics** | ***S.***  ***anginosus*** | | | ***S.***  ***agalactiae*** | | | ***S. constellatus*** | | | ***S. mitis/***  ***S. oralis*** | | | ***S. pneumoniae*** | | |
| --- | --- | --- | --- | --- | --- | --- | --- | --- | --- | --- | --- | --- | --- | --- | --- |
|  | **CI** | **10 h** | **16**  **h** | **CI** | **10 h** | **16 h** | **CI** | **10 h** | **16 h** | **CI** | **10 h** | **16 h** | **CI** | **10 h** | **16 h** |
| **Ampicillin** |  |  |  |  |  |  |  |  |  | I | S |  |  |  |  |
| **Ceftriaxone** |  |  |  |  |  |  | I | S |  |  |  |  |  |  |  |
| **Linezolid** |  |  |  |  |  |  |  |  |  | S | S | I |  |  |  |
| **Moxifloxacin** | I,S | R,S | R,R |  |  |  |  |  |  |  |  |  |  |  |  |
| **Penicillin** |  |  |  |  |  |  | I | S |  |  |  |  |  |  |  |
| **Tetracycline** |  |  |  | S | R | S |  |  |  |  |  |  |  |  |  |
| **Trimethoprim/ Sulfamethoxazole** |  |  |  |  |  |  |  |  |  |  |  |  | S | I |  |

 Highlighted orange= major error

**Supplemental Table 4.** Discordant AST results for Streptococcus species comparing MLA incubation at 10 h and 16 h versus conventional incubation (CI), with major errors indicated.
