# Supplementary material for: Optimization of culture read times using full microbiology laboratory automation
Source: J Clin Microbiol. 2026 Sep 18;64(10):e00232-26. doi: 10.1128/jcm.00232-26 (PMC13712942; doi:10.1128/jcm.00232-26)
Supplement: Supplementary file 5 [file jcm.00232-26-s0005.docx]

**Supplemental Table 5. Discordant AST results for Gram-negative Bacteria at MLA Incubations of 10 h and 16 h Compared CI Routine Incubation Time**

| **Antibiotics** | ***Acinetobacter***  ***spp.*** | | | ***E. cloacae***  ***complex*** | | | ***E.***  ***coli*** | | | ***Klebsiella***  ***spp.*** | | | ***P.***  ***aeruginosa*** | | | ***P.***  ***mirabilis*** | | | ***S.***  ***marcescens*** | | |
| --- | --- | --- | --- | --- | --- | --- | --- | --- | --- | --- | --- | --- | --- | --- | --- | --- | --- | --- | --- | --- | --- |
|  | **CI** | **10 h** | **16 h** | **CI** | **10 h** | **16 h** | **CI** | **10 h** | **16 h** | **CI** | **10 h** | **16 h** | **CI** | **10 h** | **16 h** | **CI** | **10 h** | **16 h** | **CI** | **10 h** | **16 h** |
| **Ampicillin/**  **Sulbactam** | I | R | R |  |  |  |  |  |  | I | R | R |  |  |  |  |  |  |  |  |  |
| **Cefazolin** |  |  |  |  |  |  | R | I | R | S, I | R, I | R,R |  |  |  | I | I | R |  |  |  |
| **Cefepime** |  |  |  | I | I | R |  |  |  | R | I | R | I | S | I | I | R | I |  |  |  |
| **Ceftazidime** |  |  |  |  |  |  |  |  |  | I | R | R | R, I | R, I | I, S |  |  |  |  |  |  |
| **Ceftriaxone** |  |  |  |  |  |  |  |  |  |  |  |  |  |  |  |  |  |  | S | R | S |
| **Cefuroxime** |  |  |  | I | S | I |  |  |  | I | R | I |  |  |  |  |  |  |  |  |  |
| **Ciprofloxacin** |  |  |  |  |  |  |  |  |  | S | I | I |  |  |  |  |  |  |  |  |  |
| **Ertapenem** |  |  |  | S, I | I, I | R,R |  |  |  |  |  |  |  |  |  |  |  |  |  |  |  |
| **Meropenem** |  |  |  |  |  |  |  |  |  |  |  |  | I | S | I |  |  |  | R | I | I |
| **Nitrofurantoin** |  |  |  | S, I | I, S | I, S | I | S | I | R, R, R, I | I, I, R,S | I, R, S, S |  |  |  |  |  |  |  |  |  |
| **Piperacillin/**  **Tazobactam** | I,   S, S | S,  S,S | I,   I, I |  |  |  |  |  |  | I | R | R | R | S | I |  |  |  |  |  |  |

 Highlighted orange= major error and yellow= very major error

**Supplemental Table 5**. Discordant AST results for Gram-negative bacteria comparing MLA incubation at 10 h and 16 h versus conventional incubation (CI), with major and very major errors indicated.
